# Sexual Minority Status, Anxiety–Depression, and Academic Outcomes: The Role of Campus Climate Perceptions among Italian Higher Education Students

**DOI:** 10.3390/bs10120179

**Published:** 2020-11-26

**Authors:** Anna Lisa Amodeo, Concetta Esposito, Camilla Esposito, Dario Bacchini

**Affiliations:** 1 Department of Humanistic Studies, University of Naples “Federico II”, 80133 Napoli, Italy; amodeo@unina.it (A.L.A.); dario.bacchini@unina.it (D.B.); 2 SInAPSi Center, University of Naples “Federico II”, 80133 Napoli, Italy; camillaesposito1994@gmail.com

**Keywords:** LGBQ+, campus climate, higher education, anxiety–depression, academic success

## Abstract

Students from sexual minorities generally describe Higher Education contexts as unwelcoming and chilly environments. Based on the Minority Stress theory, these disparities in climate perceptions may lead sexual minority students to negative health and academic outcomes. To date, research documenting the experience of sexual minority students within European Higher Education Institutions is limited. Framed within campus climate literature, the current study aimed to expand on previous knowledge by investigating the associations between sexual minority status, students’ perceptions of campus climate and psychological (i.e., anxiety–depression), and academic outcomes (i.e., intellectual and academic success and considering leaving the university) using a self-selected sample of 868 Italian university students (17.9% sexual minority students). The results showed that sexual minority status was associated with negative perceptions of campus climate, which, in turn, were associated with higher levels of anxiety–depression symptoms, lowered academic success, and a high probability of considering leaving university. Further research is needed to investigate the experience of sexual minority students within European Higher Education contexts and to explore possible actions that could contribute to fostering a greater sense of belonging to the campus community for all students, and particularly for students from sexual minority groups.

## 1. Introduction

Sexual minorities are a group whose sexual orientation, gender identity, and/or expression differ from the heteronormative ones [1]. Usually, sexual minorities include lesbian, gay, bisexual, transgender, and genderqueer individuals. In Higher Education contexts, sexual minority students often face unique challenges. This has been well documented in a consistent body of large national studies carried out in the United States (US) [2]. Most of these studies showed sexual minorities as the least accepted groups when compared with other under-served populations and, consequently, more likely to report deleterious experiences and unwelcoming campus climates based on sexual and gender identity. Stress, relationship difficulties, and campus climate might interfere with a successful university experience and development, independent of one’s gender or sexual identity status. However, students who identify as homosexual, bisexual, or gender diverse, may encounter the additional stress related to being part of a sexual minority group, as it has been theorized within the Minority Stress framework [3], that could lead them to several negative outcomes.

Although there has been increasing attention to equality and diversity in European universities, mainly dictated by resolutions of the European Parliament that oblige Member States to adjust their national laws to ensure adequate protection of sexual minority civil rights [4], still little is known about campus climate for sexual minority students in European Higher Education contexts. Indeed, while there is an overwhelming body of literature documenting the experience of college and university students in the US, in Europe, empirical research on prejudice and discrimination against sexual minority students within Higher Education contexts remains limited, with only a few examples coming from the United Kingdom (UK) [5] and, more recently, from Italy [6]. Overall, these studies suggested that students from these two countries consider their universities as not welcoming environments for sexual and gender minorities. Using a sample of Italian Higher Education students, the current study aimed to examine how students from sexual minority groups experience campus climate, compared to non-sexual minority students, and how these perceptions are associated with mental health problems (i.e., anxiety–depression symptoms), and academic outcomes (i.e., intellectual and academic success and thinking about leaving university).

### 1.1. Minority Stress Framework

Numerous studies have shown that members of minority groups experience disproportionate amounts of psychological distress and disorders compared to the general population [7]. The Minority Stress theory [3] states that sexual minorities, as well as all minority groups, experience chronic stress caused by continuous social stigmatization so that any social condition characterized by prejudice and discrimination is a stressor. According to Meyer [8], minority stress has three characteristics. It is (1) unique, since it is an additive factor to the general stress factors experienced by all people, requiring greater effort to adapt to members of groups that suffer stigmatization; (2) chronic, since it depends on relatively stable socio-cultural structures; (3) socially based, since it derives from social, structural and institutional processes, independent of the individual. Borrowing Lazarus and Folkman’s conceptualizations of the distal–proximal dimension of social structures [9], Meyer conceptualized distal and proximal stress processes. Distal processes refer to objective stressors independent of the individual (e.g., prejudice events), whereas proximal processes refer to stressors that are linked to the individual feelings, thoughts, and actions, as well as to his/her subjective perceptions and evaluations (e.g., internalized homophobia). Such stressful situations have been demonstrated to negatively impact one’s physical and mental health [10,11,12]. Grounded in the Minority Stress framework, several studies have shown that experiences of discrimination and stigma are significantly associated with symptoms of depression and social anxiety among sexual minorities [11,13]. The minority stress model has also been applied to college students from sexual minority groups [14,15], although research in Higher Education contexts remains scarce [16]. Scholars have found significant detrimental consequences associated with overt instances of harassment and violence experienced by lesbian, gay, bisexual, queer, and other sexual minority students (LGBQ+). LGBQ+ individuals on campuses, but also to subtler forms of heterosexism and homo-bi-transphobic discrimination that insidiously shape the social and academic experience of LGBQ+ students [2,17,18]. Findings from Woodford and Kulick [14], for instance, highlighted that heterosexism on campus was associated with decreased academic and social integration among sexual minority college students. Furthermore, personal heterosexist harassment was positively associated with academic disengagement and negatively with grade point average. Similarly, Dunbar et al. [19] found that sexual minority students endorsed significantly higher rates of psychological distress and mental health-related academic impairment compared to non-minority students.

### 1.2. Campus Climate and Negative Outcomes in Sexual Minority Students

How individuals and groups experience membership in the campus community has been generally defined as “campus climate” [2]. A consistent body of research has highlighted that a chilly climate within academic contexts, as opposed to an inclusive and welcoming climate, significantly impacts students’ psychological health and academic progress [14,20]. This finding is consistent with Bronfenbrenner’s ecological model [21], according to which individuals’ interactions with their multiple environments help them make sense of the world around them. Overall, students who are engaged with the college, either through positive faculty–student interactions, involvement in student clubs, or participation in collaborative learning projects in the classroom, are more likely to perceive their experience on campus as positive and to be retained [22,23].

This issue is of crucial concern when examining campus climate for minority groups, such as LGBQ+ students. Indeed, although the visibility of LGBQ+ people on campuses during the last two decades has largely increased [24], heterosexist and cisgender beliefs that people naturally engage only in relationships with people of the other sex and assume distinct gender roles, respectively, remain prevalent in educational settings [25]. Prior research suggests that sexual and gender minorities often report overt experiences of intimidation, harassment, and violence in academic environments [17,25,26]. Overall, they tend to perceive the university campus climate as being more hostile and dangerous than their non-LGBQ+ peers do [15,27]. In the study by Yost and Gilmore [27], half of the LGBQ+ students reported having experienced verbal harassment on campus, and 10% were victims of physical threats or attacks because of being perceived as LGBQ+. The study by Norris et al. [25] indicated that 52% of heterosexual students and 72% of sexual minority students reported hearing students make sexual orientation-based slurs sometimes or often, whereas 19% of heterosexual students and 42% of sexual minority students heard faculty make such slurs at least rarely. In a sample of Italian university students, Amodeo et al. [6] found that most participants (66.6%) reported “occasionally to very frequently” to have been witness to heterosexist microaggressions on campus.

According to the minority stress framework, such disparities in campus climate perceptions may constitute a unique, chronic, and institutionalized stressor for minority students, that put them at higher risk of poorer mental health [15] and academic outcomes [17,26]. However, only a few studies, to date, have investigated the associations between negative perceptions of campus climate and students’ outcomes. Rankin and colleagues (2010), for instance, found that sexual minority students reported higher levels of depression and slightly lower levels of perceived social support. Furthermore, about 25% of interviewed LGBQ+ students felt uncomfortable with their campus’ overall climate, whereas 28%, which was a higher percentage than among heterosexual students, seriously considered transferring to another college or university. Nevertheless, these associations between comfort with campus climate and negative outcomes were not statistically tested. Overall, research that focused on the persistence and/or retention of minority students highlighted the importance of campus climate. In a recent study by Morris and Lent [28], the experience of heterosexist harassment was a risk factor for college withdrawal or transfer. That is, students who experienced harassment were somewhat less likely to want to remain at their campuses, possibly because of perceptions of limited social acceptance or academic disengagement. Similarly, Blumenfeld, Weber, and Rankin [29] found that respondents attending unwelcoming and hostile campuses reported lower interest in remaining at their current campuses and discouraged future students, staff, faculty, and administrators from attending.

Several scholars have pointed to intellectual and academic success as a key factor for student retention in Higher Education [30]. To date, only a few studies have examined how campus climate for LGBQ+ ties with academic success [31]. Based on Tinto’s Integration Theory [32], students’ academic success can be defined in terms of commitment they have to their academic and career goals, as well as to the institution. In a recent study by Garvey et al. [31], sexual minority students who had more positive perceptions of institutional action regarding campus climate and warmer perceptions of campus climate rated their academic success as higher. Furthermore, there is still little evidence about whether sexual minority students feel that their academic experiences and satisfaction with their intellectual growth are affected by their sexual identity. Further investigation of this topic is needed.

### 1.3. The Present Study

To date, the literature documenting the experience of sexual minority students within European Higher Education Institutions is limited. According to the most recent ILGA-Europe (the International Lesbian, Gay, Bisexual, Trans and Intersex Association; [33]) report, Italy is among the European countries with the highest level of discrimination and the worst legal and policy situation for sexual minorities. The legal recognition of same-sex civil unions and unregistered cohabitation was legally recognized only in 2016, but reproductive rights for same-sex partners are not covered [34]. Furthermore, specific legislation to punish hate crimes perpetrated against sexual minorities is still debated. The need to devote special attention to the Italian context is also supported by evidence from the scientific literature. Overall, Italian sexual minorities have frequently been found to face heterosexist prejudices in their daily lives [31], and negative attitudes toward same-sex parenting are still widespread. Research about negative attitudes toward homosexuality in Italy shows that Italian people are quite ambivalent toward homosexuality [35,36]. On the one hand, homosexuality is considered as a sin or a deviation from normal development; on the other hand, it is considered as a private matter, something that should be neither persecuted nor protected by law.

**Figure 1:**
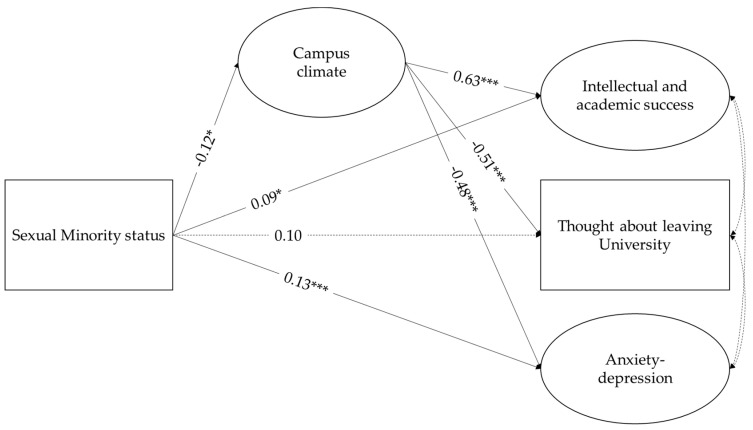
The associations between sexual minority status, campus climate perceptions, and health and academic outcomes. Higher scores of campus climate reflect more positive evaluations. *** *p* < 0.001, * *p* < 0.05.

To our knowledge, the recent study by Amodeo et al. [6] was the first to focus on negative perceptions of campus climate in a sample of Italian Higher Education students. More specifically, the authors investigated how heterosexist environmental microaggressions taking place on campus contributed to the negative evaluation of campus climate for both heterosexual and non-heterosexual students. The current study is framed through the theoretical body of literature examining campus climate. Studies of campus climate for LGBQ+ students have generally focused on three areas: (a) perceptions and experiences of LGBQ+ students, (b) perceptions about LGBQ+ students and their experiences, and (c) the status of policies and programs designed to improve the academic and living experiences of LGBQ students on campus [37]. In the current study, campus climate has been conceptualized as measurable perceptions of various aspects of university life: General climate for diversity, referring to perceptions relative to the overall comfort that students perceive with the climate for diversity at their university; and LGBQ+ campus climate, referring to the specific perceptions about the degree to which the general atmosphere on campus is accepting and supportive of LGBQ+ identities. This study aims to expand on previous knowledge by investigating the associations between sexual minority status, students’ perceptions of campus climate, and psychological (i.e., anxiety–depression) and academic outcomes (i.e., intellectual and academic success and considering leaving the university) using a sample of Italian university students (Figure 1). More specifically, we hypothesized that sexual minority students were more likely to report negative evaluations of campus climate, compared to non-minority students. Furthermore, we expected that they also reported higher levels of anxiety–depression, lower scores of intellectual and academic success, and were more likely to consider leaving their university (Hypothesis 1). We expected that negative campus climate perceptions were associated with higher anxiety–depression, lower intellectual and academic success, and a higher probability of considering leaving university (Hypothesis 2). Finally, we tested whether these associations were mediated by negative perceptions of campus climate(Hypothesis 3). Students’ biological sex, age, yearly income, and level of participation in university activities were used as control variables.

## 2. Materials and Methods

### 2.1. Participants and Procedure

Data were collected in 2019 through an anonymous web-based survey of students from a large university in Southern Italy. This initiative was launched by the Anti-discrimination division of the University Service Center (SInAPSi—Center for Active and Participatory Inclusion of Students) as a means to gauge students’ perceptions of the actual campus climate for LGBQ+ and design, based on this information, policies and programs to create a climate that better aligns with the university mission and values. The study conformed to the principles of the Declaration of Helsinki on Ethical Principles for Medical Research Involving Human Subjects and was approved by the University Institutional Review Board (project identification code: 35/2019). Students were invited to complete the survey by faculty engaged in the institutional committee for Diversity, Equity, and Inclusion, who were contacted by staff working at the Anti-discrimination division of the University Service Center. A snowball sampling procedure was used, asking students to share the survey with any other students they knew at the same university. Privacy was guaranteed to participants in accordance with Italian laws 196/2003 and 101/2018. Informed consent was obtained before the administration of questionnaires. The participation was voluntary, and participants could withdraw at any time without any adverse consequence. The sample consisted of 1006 students attending several academic programs offered by the University. One hundred and thirty-six surveys were excluded from analysis due to missing data on all key study variables. The final sample comprised 868 Italian students (71% female). Participants were 18 to 45 years old (*M* = 21.51, *SD* = 8.07). One hundred and fifty-five (17.9%) students self-identified as non-heterosexual or non-cisgender. No participant self-identified as transgender or transsexual. Descriptive statistics of sample characteristics are reported in Table 1.

### 2.2. Measures

#### 2.2.1. Sociodemographic Characteristics and Controls

Sociodemographic variables included sex assigned at birth (male, female, intersex), actual perceived gender (man, woman, genderqueer, and other with specification required), sexual orientation (lesbian, gay, bisexual, asexual, and other with specification required), age, family yearly income. In terms of academic variables, we asked participants to indicate their level of participation in academic activities (more than 75% of planned activities, between 50% and 75% of planned activities, between 25% and 50% of planned activities, less than 25% of planned activities, non-attending student).

#### 2.2.2. Perceptions of Campus Climate

Campus climate perceptions were measured using ad hoc items that have been linked to campus climate in prior literature [16]. More specifically, participants were asked to indicate how comfortable they personally felt with the climate for diversity at their university. Furthermore, they were asked to rate how welcoming and respectful they perceived their academic institution with respect to (i) gay men, (ii) lesbian women, (iii) bisexual people, (iv) gender diverse people. Responses were collected on a 5-point scale ranging from 1 to 5, with higher values indicating more positive evaluations. Cronbach’s alpha for the global measure was 0.89. One latent factor was derived as a global measure of campus climate.

#### 2.2.3. Anxiety–Depression

Symptoms of anxiety–depression were measured by using the Adult Self-Report (ASR) [38]. The overall questionnaire consists of 123 items assessing internalizing and externalizing dimensions of problem behavior (e.g., aggression, withdrawal, somatic complaints). For the current study’s purposes, the specific anxiety–depression subscale was used, consisting of 17 items (e.g., I feel lonely; I feel worthless or inferior). Each item was scored on a three-point scale (0 = “not true”, 1 = “somewhat or sometimes true”, and 2 = “very true or often true”). The Italian translation of the questionnaire has been used in prior studies [39], demonstrating adequate reliability and cross-cultural consistency. The reliability of the measure in this study’s sample was good (Cronbach’s alpha = 0.91).

#### 2.2.4. Academic Outcomes

Six items from the academic and intellectual development scale developed by Pascarella and Terenzini [40] were used to measure the degree of students’ academic success. Items from this scale have been used in prior studies to assess students’ academic experience [31]. Sample items were “I am satisfied with the extent of my intellectual development since enrolling in this university” and “My academic experience has had a positive influence on my intellectual growth and interest in ideas”. Items were translated from English into Italian by two native Italian speakers, experts in psychology and fluent in English. Two different versions were obtained and compared, achieving a final agreement. Then, an American native English speaker translated the obtained version from Italian to English to confirm that the translation was accurate. The scale ranged from “strongly disagree” (1) to “strongly agree” (5), with higher values indicative of greater intellectual and academic success. The confirmatory factor analysis confirmed the psychometric structure of the scale, χ^2^ (8) = 61.21, *p* < 0.001, CFI (Comparative Fit Index) = 0.98, RMSEA (Root Mean Square Error of Approximation) = 0.08, SRMR (Standardized Root Mean Square Residua) = 0.02. The reliability coefficient was adequate, Cronbach’s alpha = 0.85.

In addition, participants were asked whether they had considered leaving their university in the last 12 months. Responses options for this question were “yes” or “no”.

### 2.3. Statistical Analysis

The study’s hypotheses were tested using structural equation modeling (SEM) in Mplus version 8 [41]. Analyses were performed using the Weighted Least Square Mean and Variance Adjusted chi-squares (WLSMV) estimator, which is the recommended estimator when continuous and categorical dependent variables are included in the same model [42]. Three latent factors were estimated as part of the main structural equation model. Two factors were created to reflect academic success and anxiety–depression, respectively. The other one was derived from two observed indicators: One that reflected the individual overall perception of comfort with campus climate, the other one reflecting student perceptions of campus climate for LGBQ+ people. A series of confounding variables were considered in the study: biological sex (male vs. female), age, yearly income, and level of academic participation. Multiple fit indices were used to evaluate model fit: chi-square likelihood ratio statistic, CFI, RMSEA with associated 90% C.I., and SRMR. Guided by suggestions provided by Hu and Bentler [43], the following criteria were used to identify an acceptable model fit: CFI ≥ 0.95, RMSEA ≤ 0.06, and SRMR < 0.05.

We initially tested the main effect of sexual minority status on campus climate perception, anxiety–depression symptoms, intellectual and academic success, and having thought about leaving university (Hypothesis 1). Then, we examined how campus climate was associated with anxiety–depression symptoms, intellectual and academic success, and having thought about leaving university (Hypothesis 2). Finally, we examined whether campus climate perceptions mediated the relationship between sexual minority status, on one side, and anxiety–depression symptoms, intellectual and academic success, and having thought about leaving campus, on the other side (Hypothesis 3). The mediation effects were tested using bias-corrected bootstrap confidence intervals based on 1000 resamples. Confidence intervals that do not contain zero indicate a significant indirect effect via the specific mediator.

## 3. Results

### 3.1. Descriptive Statistics and Bivariate Correlations

Bivariate correlations between all variables included in the study are shown in Table 2. As can be observed, positive perceptions of campus climate were significantly associated with higher academic success and lower anxiety depression symptoms, and vice-versa. Students who reported having thought about leaving their university also reported low academic success, negative perceptions of campus climate, and higher symptoms of anxiety–depression, compared to those who did not report having thought about leaving their university. 

Anxiety–depression was negatively related to positive campus climate perception and academic success. With respect to control variables, family yearly income was negatively associated with anxiety–depression and having thought about leaving university. Higher levels of participation in academic activities were associated with negative perceptions of campus climate. Biological sex only significantly correlated to anxiety–depression, with females being more likely to report anxiety–depression symptoms.

### 3.2. Sexual Minority Status, Perceptions of Campus Climate and Negative Outcomes

Results of the structural equation modeling are shown in Figure 1. The model showed an adequate fit to the data, χ^2^ (88) = 337.61, *p* < 0.001, CFI = 0.95, RMSEA = 0.06 with 90% C.I. [0.05,0.06], SRMR = 0.04. Overall, sexual minority status was significantly associated with campus climate perception and anxiety–depression, with LGBQ+ perceiving campus climate more negatively than their counterparts and reporting higher scores of anxiety–depression. Furthermore, sexual minority status was associated with higher levels of academic success. No significant direct association was found between sexual minority status and having thought about leaving university (Hypothesis 1). Negative perceptions of campus climate were linked to lower academic success, higher anxiety–depression symptoms, and a greater probability of having thought about leaving university (Hypothesis 2). The mediation analysis highlighted marginal significant indirect effects for each of the outcome variables considered in the study (Hypothesis 3; anxiety–depression, b = 0.06, *p* < 0.05, 95% C.I. [0.001,0.13]; intellectual and academic success, b = −0.08, *p* < 0.05, 95% C.I. [−0.244,−0.003]; leaving campus, b = 0.06, *p* < 0.05, 95% C.I. [0.002,0.165]). With respect to control variables, participation in academic activities was significantly related to negative campus climate perceptions. Biological sex had a significant effect on anxiety–depression, with females reporting higher levels of symptoms. Finally, having thought about leaving university was significantly associated with low family yearly income. For each outcome, a significant percentage of variance was explained (38% for intellectual and academic success, 27% for having thought about leaving university, and 29% for anxiety–depression).

## 4. Discussion

Research on campus climate at colleges and universities has largely increased in the US during the last decades [29], consistently indicating that sexual prejudice in Higher Education settings is a significant problem. In Italy, as well as in many other European countries, studies documenting how LGBQ+ students and staff experience university environments, how they feel on campus, and how academic experiences are associated with mental health and academic success remain limited.

The current study aimed to investigate the associations between sexual minority status, students’ perceptions of campus climate and psychological (i.e., anxiety–depression) and academic outcomes (i.e., academic success and considering leaving university) in a sample of Italian university students. Based on prior literature from US campuses, we hypothesized that sexual minority students were more likely to report negative evaluations of campus climate compared to non-minority students. Furthermore, we expected that they would also report higher levels of anxiety–depression, lower scores of academic success, and a high likelihood of considering leaving their university (Hypothesis 1). Campus climate perceptions were expected to be associated with all outcomes considered in the study (Hypothesis 2). Finally, we tested whether the associations between sexual minority status and anxiety–depression, academic success, and having thought about leaving university were mediated by negative perceptions of campus climate (Hypothesis 3). Overall, the results of the study partially supported our hypotheses, indicating that sexual minority groups had more negative perceptions of campus climate and higher levels of anxious–depressive symptomatology. Surprisingly, sexual minority students reported higher levels of academic success compared to their non-sexual minority peers, whereas no significant direct relationship was found between a student’s sexual minority status and whether the student had thought about leaving the university. With respect to the mediation analysis, the findings supported the hypothesis that campus climate perceptions significantly mediated the relationships between sexual minority status and all outcomes considered in the study.

In regard to the association between sexual minority status and campus climate, we found that LGBQ+ students reported higher negative perceptions of campus climate. This result is in line with several previous studies carried out in the US Higher Education settings [27,44,45,46] and could be interpreted in light of the considerable levels of sexual prejudice and heterosexism that have been documented in the limited previous research involving Italian university students [6,35]. Contrasting discrimination on the grounds of gender and sexual orientation has become a crucial part of European Union (EU) policies and the subject of numerous resolutions of the European Parliament, thus obliging Member States to adopt relevant anti-discrimination legislation. However, important fields, such as social protection, education, and access to goods and services, are not fully covered by these provisions, leaving sexual minorities particularly vulnerable in these areas. With the introduction of the Employment Equality Regulations [47], European universities were required, for the first time, to ensure that staff and students are not discriminated against based on sexual orientation. Since then, considerable attention at the national level has been devoted to developing policies that allow for equal rights for LGBQ+ students and staff and address discrimination issues within Higher Education contexts. However, these policies are rarely successfully implemented, despite that conditions related to campus climate, when examined, appear critical for LGBQ+ students.

Findings of the current study also showed that sexual minority students reported significantly higher levels of anxiety–depression. Prior studies using population-based sampling have shown that sexual minorities experience at least a two-times greater likelihood of major depressive disorder and anxiety disorders compared to non-sexual minorities [48,49,50]. Using a sample of university students in the US, Dunbar et al. [19] found that LGBQ+ students were more likely than non-LGBQ+ peers to endorse serious psychological distress and emotional and behavioral issues that affected their academic impairment, consistent with previous empirical evidence [51]. Using the minority stress model as an interpretative framework [3], the literature has largely recognized these mental health disparities as a consequence of sexual minorities’ disproportionate exposure to stigma-related stress, compared to non-sexual minority groups.

Surprisingly, we found that sexual minority students scored marginally higher, on average, on intellectual and academic success, compared to their non-sexual minority peers. To our knowledge, there are no previous studies comparing experiences of academic success in sexual minority and non-sexual minority students. Overall, this finding suggests that LGBQ+ students might experience positive intellectual and academic growth independent of their general campus climate perception. The study by Blumenfeld et al. [29] would support this hypothesis, as it showed that a significant number of LGBQ+ respondents were satisfied and comfortable within their individual academic or work departments or programs of study, although they reported discomfort with the overall campus climate. Future studies should further deepen these aspects of students’ academic experience, such as faculty–student interactions and academic programming, and explore how they relate to students’ sexual minority status and academic success.

No significant direct relationship was found between sexual minority status and whether the student had thought about leaving university, thus suggesting that both LGBQ+ and non-sexual minority students are at similar risk for dropping out. As expected, campus climate perceptions were related to all outcomes considered in the study. In line with the previous empirical evidence, negative perceptions of campus climate, independent of sexual minority status, were associated with a higher risk for anxiety–depression, a higher probability of considering leaving university, and lower intellectual and academic success. More interesting, when examining indirect effects, the results highlighted that campus climate perceptions significantly mediated the effect of sexual minority status on anxiety–depression, having thought about leaving university, and academic success. That is, LGBQ+ students had more negative perceptions of campus climate, which in turn, put them at higher risk for anxiety–depression and leaving their university, and more negative evaluations of their academic and intellectual development. Overall, these results are consistent with previous literature indicating significant relationships between LGBQ+ campus climate perceptions and mental health problems [26], thoughts about leaving university [29], and academic success [14,31] in samples of sexual minority students. Taken together, such findings support the hypothesis that LGBQ+ students perceive their university environment as more hostile and less welcoming for non-sexual minority students, and this may lead them to develop a series of negative outcomes, including mental health problems, decreased interest in remaining at their current university, and decrements in academic success.

The findings of the current study should be considered in light of several limitations. First, data were collected from a small sample of students, mostly female and non-sexual minority students, and all coming from one Higher Education Institution in Italy. While focusing on campus climate for sexual minorities in Italy represents an important contribution to the scientific literature, a more robust investigation on a large national scale would be needed for the generalization of results. Furthermore, given the use of a cross-sectional design, the hypothesized causal relationships between the study’s variables cannot be statistically determined. One possible alternative model could be that negative perceptions of campus climate might depend on students’ high levels of anxiety–depression and/or low academic outcomes, and not vice versa, as hypothesized in the current model. However, it might also be plausible that bidirectional associations could better explain the relationship between these constructs. Longitudinal research considering at least two measurement points is needed to clarify these relationships. Furthermore, it is important to note that, while significant, the statistical effects reported in the study tended to be quite small, as indicated by low significance levels of *p*-values and bootstrap confidence intervals near to zero for mediation effects. Future studies should consider several other dimensions of the campus climate measure, such as the perception of institutional support, academic programming, or other aspects related to the experiential climate (e.g., heterosexist microaggression). Future research should also investigate potential differences among sexual minority subgroups that were not considered in the current study due to the limited sample size. Some studies, for instance, have highlighted that bisexual individuals experience additional stressors related to their sexual identity compared to lesbians and gay men. Bisexual people are often perceived as sexually irresponsible, promiscuous, or unable to have monogamous relationships, even by other sexual minorities, which could lead them to more negative experiences within the academic context. In light of the above considerations, caution is needed when interpreting the results.

## 5. Conclusions

Campus climate issues represent a crucial part of the agenda for Higher Education Institutions due to the relevant implications that negative experiences within academic contexts have on individual psychological health and academic achievements. Creating environments that welcome, support, and promote diversity is among the main challenges of Higher Education in the 21st Century. In contrast to the US, where assessing campus climate is prioritized on a national scale, this remains an unmet urgent need in Europe. In 2019, the European Commission co-funded a three-year project devoted to developing and implementing an innovative tool that will support European Higher Education Institutions in evaluating the actual level of inclusiveness of their environment and in identifying any efforts that can be put in place to best address the needs of sexual and gender marginalized groups and ensure the protection of their fundamental rights and academic opportunities. A consortium of seven partners from five European countries (Italy, Ireland, Slovenia, Greece, and Spain) is currently working on the development of the index (namely, the XENIA Index; www.xeniaindex.eu), that will be delivered and made available for all European Higher Education Institutions by the end of 2022. Further studies should continue to document the experience of sexual minority groups within European Higher Education contexts and explore possible actions that could foster a greater sense of belonging in the campus community for all students, particularly for students from sexual minority groups.

## Figures and Tables

**Table 1:** Descriptive statistics of the sample.

Characteristics	*N* = 868 *N* (%)
Sexual orientation	
LGB+	151 (17.5)
Other	717 (82.5)
Gender identity	
Cisgender	855 (98.5)
Genderqueer	13 (1.5)
Family yearly income	
≤15,000	436 (50.2)
16,000≤ ≥50,000	347 (40)
≥51,000	85 (9.8)
Participation in academic activities	
more than 75% of planned activities	575 (66.3)
between 50% and 75% of planned activities	201 (23.1)
between 25% and 50% of planned activities	53 (6.1)
less than 25% of planned activities	26 (3.0)
non-attending student	13 (1.5)

**Table 2:** Correlations among study’s variables.

	1	2	3	4	5	6	7	8	9
1. Family yearly income	1								
2. Age	0.07 *	1							
3. Biological sex (Male)	0.07 *	0.18 ***	1						
4. Participation in academic activities	−0.02	0.18 **	−0.05	1					
5. Sexual Minority status (LGBQ+)	−0.06	−0.09 **	0.04	0.04	1				
6. Campus climate	0.03	−0.02	0.02	−0.23 ***	−0.13 *	1			
7. Intellectual and academic success	0.04	0.04	−0.01	−0.1	0	0.60 ***	1		
8. Having thought about leaving university (Yes)	−0.11 **	0.05	0.02	0.07	0.11 **	−0.51 ***	−0.26 ***	1	
9. Anxiety–depression	−0.07 *	0	−0.20 ***	0.05	0.18 ***	−0.48 ***	−0.27 ***	0.35 ***	1

*** *p* < 0.001, ** *p* < 0.01, * *p* < 0.05.
